# Use of genetically encoded, light-gated ion translocators to control tumorigenesis

**DOI:** 10.18632/oncotarget.8036

**Published:** 2016-03-16

**Authors:** Brook T. Chernet, Dany S. Adams, Maria Lobikin, Michael Levin

**Affiliations:** ^1^ Center for Regenerative and Developmental Biology and Department of Biology Tufts University, Medford, MA 02155, USA

**Keywords:** V_mem_, bioelectricity, voltage, RAS, optogenetics

## Abstract

It has long been known that the resting potential of tumor cells is depolarized relative to their normal counterparts. More recent work has provided evidence that resting potential is not just a readout of cell state: it regulates cell behavior as well. Thus, the ability to control resting potential *in vivo* would provide a powerful new tool for the study and treatment of tumors, a tool capable of revealing living-state physiological information impossible to obtain using molecular tools applied to isolated cell components. Here we describe the first use of optogenetics to manipulate ion-flux mediated regulation of membrane potential specifically to prevent and cause regression of oncogene-induced tumors. Injection of mutant-KRAS mRNA induces tumor-like structures with many documented similarities to tumors, in *Xenopus* tadpoles. We show that expression and activation of either *ChR2^D156A^*, a blue-light activated cation channel, or *Arch*, a green-light activated proton pump, both of which hyperpolarize cells, significantly lowers the incidence of KRAS tumor formation. Excitingly, we also demonstrate that activation of co-expressed light-activated ion translocators *after* tumor formation significantly increases the frequency with which the tumors regress in a process called normalization. These data demonstrate an optogenetic approach to dissect the biophysics of cancer. Moreover, they provide proof-of-principle for a novel class of interventions, directed at regulating cell state by targeting physiological regulators that can over-ride the presence of mutations.

## INTRODUCTION

Recent work has highlighted the instructive roles of bioelectric signals in large-scale pattern formation during embryogenesis and regeneration [1–7]. We are beginning to understand how these ionic signals function as a layer of physiological control and how they are integrated mechanistically with biochemical and genetic pathways [8–12]. Indeed, because bioelectrical states regulate differentiation, migration, and proliferation [13–17], these physiological circuits are an ideal candidate for an important aspect of the patterning cues that go awry in cancer [18–23]. Bioelectricity has long been implicated in neoplasm [24, 25], and recent molecular efforts have focused on ion channels as important cancer targets [26–33], and ion channel drugs as a promising class of therapies [13, 34–38].

Importantly however, it is beginning to be seen that modulating carcinogenesis and metastasis is not as simple as targeting individual ion channel genes for loss- or gain-of function applications. The key parameter can be a complex, non-cell-autonomous physiological state not intrinsically limited to any one specific channel or pump [3, 39–43]. Thus, it is important to exploit amenable model systems to understand how aspects of cancer are regulated by bioelectrical states of tissues *in vivo*. However, to fully understand and exploit the control of cell functions via bioelectrical signaling requires the development of new techniques that allow ion flux to be influenced in any cell/tissue of interest with much greater spatio-temporal control than has been possible to date with pharmacological or genetic approaches.

One exciting candidate for achieving improved spatio-temporal control of ion flux is optogenetics [44–47]. Optogenetics is the expression of light-gated ion translocators and enzymes, with different kinetics and different wavelengths of activation, to control downstream processes, such as ion flux or enzyme activity. It has primarily been used as a precise tool to control neural and muscle excitation/inhibition, and to regulate biochemical processes [44, 45]. Extending the use of optogenetics to non-excitable cells [48], our lab has successfully initiated *Xenopus* tail regeneration by hyperpolarizing cells using the light-dependent H^+^ pump, Archaerhodopsin, thereby reversing the normal, age-dependent loss of regenerative ability [49]. Building on this work and recent data on the bioelectric control of tumorigenesis [43, 50], we investigated here the potential applicability of optogenetics to hyperpolarization-induced tumor suppression.

*Xenopus laevis* is a model system that facilitates the investigation of the role of bioelectric signals in morphogenesis and cellular regulation [12, 51–55]. The organism also provides a powerful model system for studying cancer [4, 50, 56–60] due to its tractability for optical, molecular, and electrophysiological studies, and to the many conserved pathways it shares with humans [61–66]. Moreover, this model system offers well characterized tumor-inducing molecular reagents (the human oncogenes *Gli1*, *Xrel3*, *p53Trp248* and *KRAS^G12D^*), whose expression results in induced tumor-like structures (ITLSs) with many of the hallmarks of mammalian tumors: increased mitotic activity, induced vasculogenesis, increased hypoxia, acidic microenvironment, disorganization of normal cell architecture, and ability to trigger an innate immune response [3, 4, 50, 67, 68].

In addition to exhibiting other classic tumor characteristics, these tumor-like structures maintain a depolarized membrane voltage. This bioelectric signature can be used to detect prospective ITLS regions before they become morphologically apparent [42]. Moreover, we have shown that this depolarization is not only a signature but is functionally required for tumorigenesis, by demonstrating that artificially hyperpolarizing oncogene-expressing cells, by expressing any of several ion channels, significantly reduces the incidence of ITLS formation despite high levels of otherwise-sufficient oncogene expression in the same tissue [3, 4].

Given the importance of bioelectric signaling, and the need for high-resolution manipulation both for the clinic and for probing the basic biology of this process, we explored whether optogenetics can be used to improve our ability to regulate, in both time and space, the bioelectric signaling that is disrupted during cancer. We hypothesized that spatio-temporally-controllable light-gated ion translocators would enable precise control over ion flux (and therefore membrane voltage (V_mem_)) in a tissue of interest, and thus we sought to extend optogenetics to the biophysical control of oncogene-dependent tumorigenesis.

To test the ability of optogenetic tools to alter endogenous V_mem_ and thereby suppress ITLS growth and promote tissue normalization, we used Archaerhodopsin (*Arch*) [69], a light-gated H^+^ transporter that hyperpolarizes cells [49, 70]. We confirm here that microinjection of *KRAS^G12D^* [71] mRNA, a tumor-inducing KRAS mutant, results in the formation of the ITLSs that we have previously shown to exhibit classic hallmarks of tumors, including histopathology, increased proliferation, lack of differentiation, attraction of vasculature, etc. [3, 4, 41]. We then demonstrate that co-injection of *Arch* mRNA and subsequent expression of *Arch* in *KRAS^G12D^*-expressing cells, followed by a 24-hour light-activation of the *Arch* transporter, reduced ITLS incidence by 32%. We also show that suppression and normalization of ITLSs are not specific to *Arch*, but can also be accomplished by injection of a different optogenetic reagent, channelrhodopsin-2 (*ChR2^D156A^*) [72]. Most interestingly, by delaying the activation of *ChR2^D156A^* until ∼ stage 35, we were able to convert fully developed ITLSs into normal cells. Thus we demonstrate the utility of optogenetics to suppress ITLS formation and to promote normalization of existing ITLSs into wildtype tissue.

## RESULTS

### Injection of *KRAS^G12D^* results in ITLS formation

**Figure 1:**
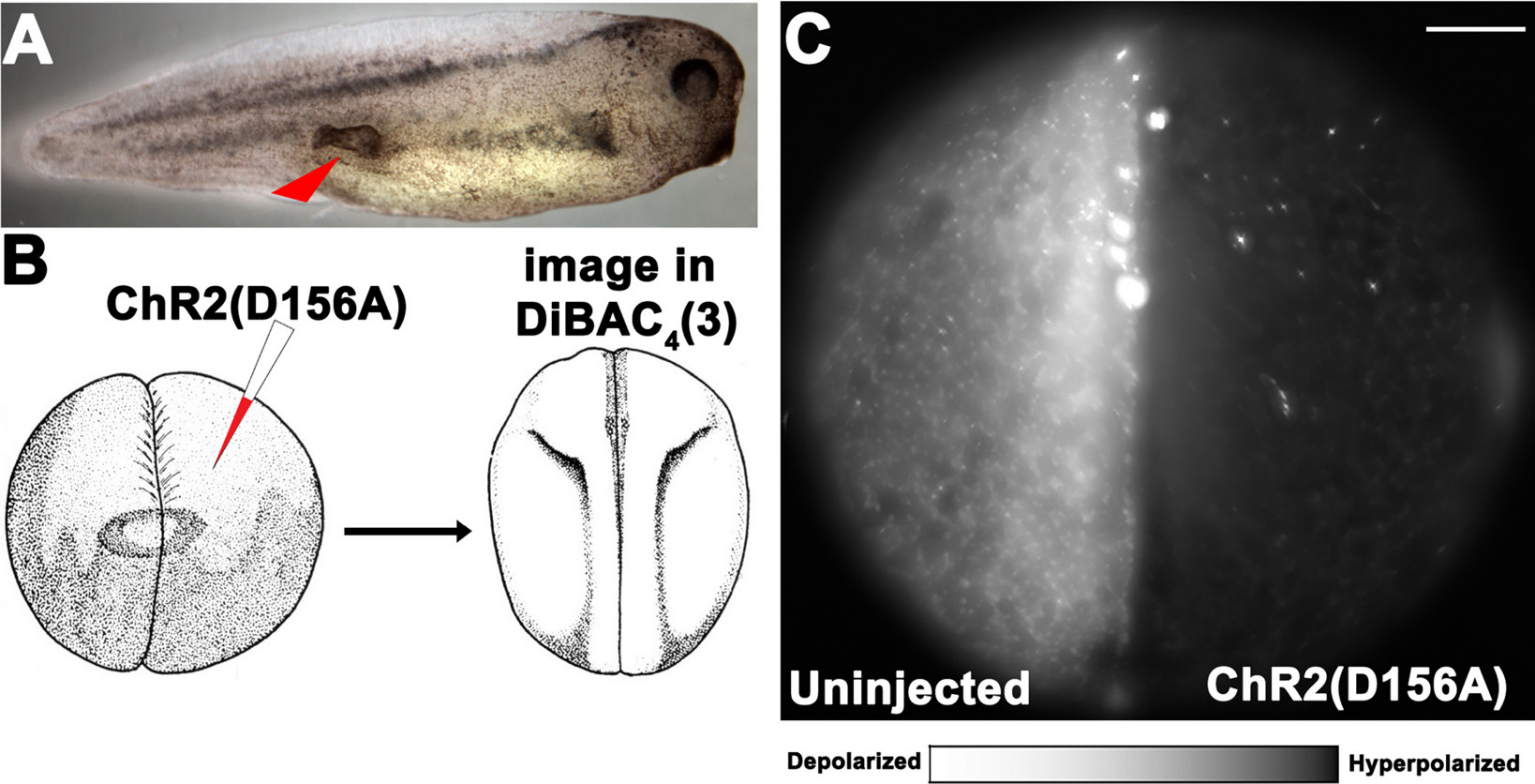
Optogenetic modulation of V_mem_ to control ITLSs is achieved using a *KRAS^G12D^* oncogene and light-sensitive ion channels in *Xenopus laevis* embryos (**A**) ITLSs were generated by injecting *KRAS^G12D^* mRNA into a single blastomere of 16 cell stage embryos. Injected embryos were raised in 0.1 × MMR before they were scored for the presence of ITLSs and imaged using bright field microscopy between stages 28 and 35. (**B**) Schematic of optogenetic V_mem_ modulation using a Channelrhodopsin-2 mutant (*ChR2^D156A^*) channel: *ChR2^D156A^* mRNA was injected into 1 cell of a 2-cell embryo, allowing the uninjected side to serve as an internal control. Embryos were raised to stage 18 in 0.1XMMR. (**C**) At stage 18, embryos were soaked in 1.9 μM DiBAC_4_(3) solution in 0.1 × MMR, and imaged using a DiBAC_4_ (3) filter set (470/20; BS 485; EM 517/23). The un-injected left half of the embryo was highly fluorescent, indicating relative depolarization compared to the right half of the embryo, which is expressing *ChR2^D156A^*. Scale bar = 150 μm.

To study the usability of V_mem_-altering optogenetic tools in oncogene-mediated tumorigenesis, we expressed a human oncogene in *Xenopus* embryos. Injection of the oncogene *KRAS^G12D^* [71] into *Xenopus* embryos (1 blastomere at the 16-cell stage) induced ITLSs (Figure 1A), which have previously been shown to exhibit many of the defining hallmarks of their mammalian counterparts.

**Figure 2:**
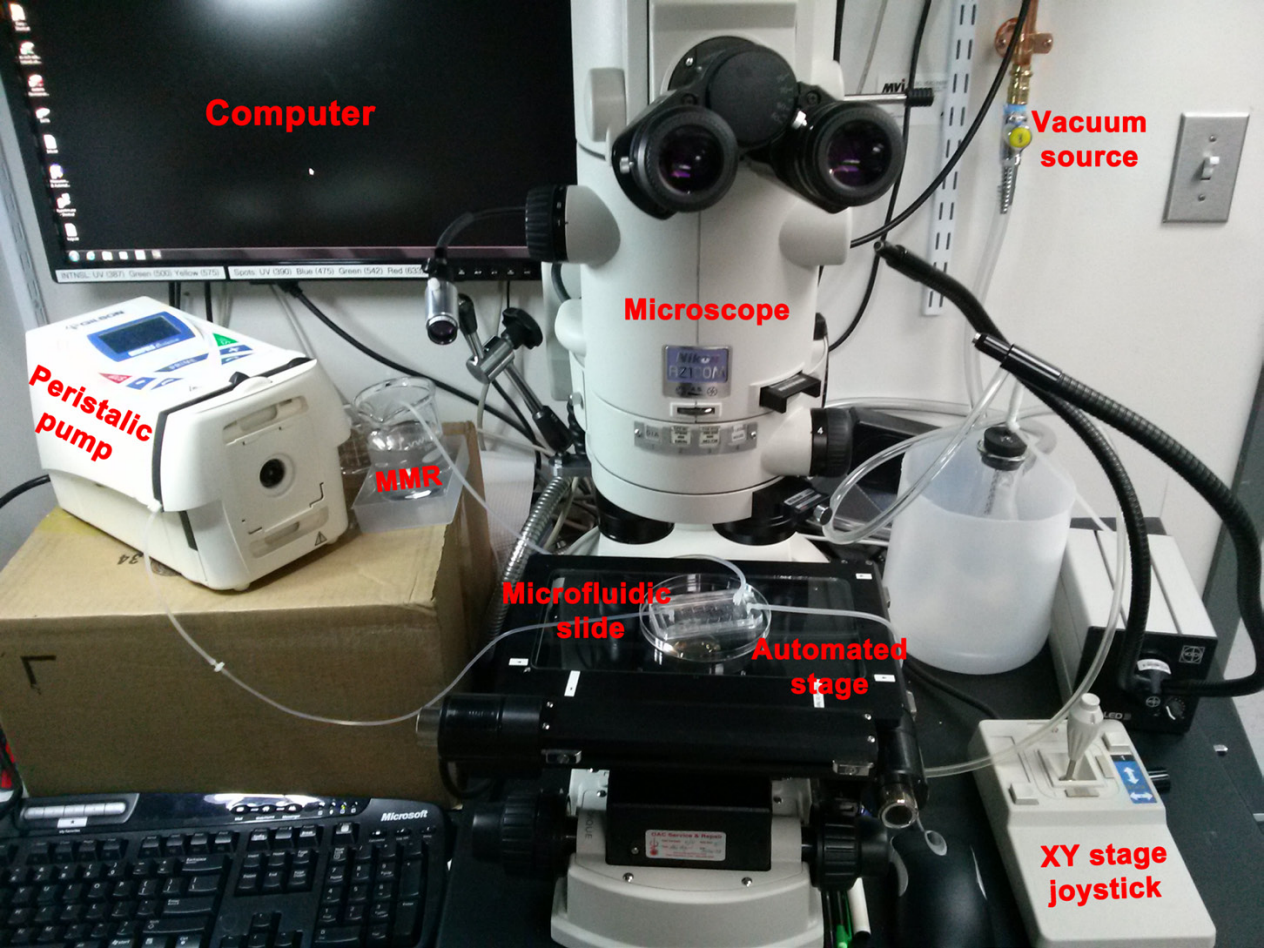
Set up used to deliver spatio-temporally precise light stimulation of optogenetic ion-translocators expressed in *Xenopus* embryo ITLSs We customized a Nikon AZ100 dissection scope for *in vivo* optogenetics [87] by replacing the epifluorescence illumination source and light guide with a Spectra4 LED illuminator connected to the scope via fiber optic cable (the black light guide that passes in front of the vacuum source). The light is passed through a pinhole to set spot size diameter (located behind the oculars, not visible in this image), then enters the scope and goes through an 80/20 splitter that allows the user to view the specimen on the monitor even while the LED is on. Finally, the light passes through the 5 × objective lens which further reduces spot diameter and aims the spot at the sample. An automated Ludl MAC6000 XY stage (that can also be manipulated manually by a joystick) allows multiple embryos to be exposed repeatedly to the activating wavelength of light. Up to thirty embryos are loaded into a slide-mounted PDMS “chip” designed to use microfluidics to hold embryos in place [100]; the chip is held to the slide by a vacuum and 0.1 × MMR is circulated by a peristaltic pump. The optogenetics components and the microscope are all controlled by NIS Elements.

### *ChR2^D156A^* activity alters membrane voltage of *Xenopus* embryonic cells

To allow modulation of V_mem_ via light activation of a channelrhodopsin-2 (*ChR2^D156A^*) channel, *ChR2^D156A^* mRNA was injected into 1 cell of a 2-cell embryo, allowing the uninjected side to serve as an internal control (Figure 1). *ChR2^D156A^* is a non-specific cation channel that, at physiological pH, passes mostly protons, but there is also significant Na^+^ and K^+^ flux [45] [73]; it was selected because of the low incidence of side effects in *Xenopus* embryos [48]. Because of the extremely low ion concentration of the surrounding medium, light activation of this channel is predicted to hyperpolarize those cells due to efflux of cations; Table 1 gives the internal and external ion concentrations. Injected embryos, raised to stage 18, were exposed to blue light then imaged using the membrane voltage-sensitive dye DiBAC_4_ (3), a semi-quantitative method that has been extensively used to monitor relative resting-potential differences among cells *in vivo* [49, 54, 74–78]. As predicted, we observed that the injected right half of the embryo was much dimmer, indicating relative hyperpolarization compared to the uninjected left side.

**Table 1:** Ion concentrations in Xenopus embryonic cells and their medium (from [102])

Ion	External medium [mM]	Intracellular [mM]
Na^+^	9.9	38
K^+^	0.2	51
Ca^++^	0.3	5
Mg^++^	0.2	12
H^+^	1.60E–05	1.80E–05
Cl^−^	11.1	30

### Arch and *ChR2^D156A^* activities reduce *KRAS^G12D^*-induced ITLS incidence

Co-injection of mRNAs for *KRAS^G12D^* and either *Arch* or *ChR2^D156A^* into one cell of a 16-cell stage embryo resulted in the expression of the corresponding proteins as early as 4 hours post injection (data not shown). Embryos expressing light-activated ion channels were exposed to light for 24 hours: arch-expressing embryos were stimulated by green light, 555 nm, irradiance of 1 mW/mm^2^ for 500 ms followed by 1.5s in darkness, while *ChR2^D156A^*-expressing cells were stimulated by blue light: 470 nm light of 2.4 mW/mm^2^ for 10 ms every 30 seconds (Figure S2, 3A; [48]). To study ITLS prevention, embryos were exposed to the light beginning four hours post injection, or, approximately stage 9; to induce normalization of tumors, exposure was from stage 28 to stage 35. Two sets of controls were used for comparison: embryos injected with only *KRAS^G12D^* and un-stimulated embryos expressing the oncogene and either *Arch* or *ChR2^D156A^* (light by itself does not affect V_mem_ of cells that do not express light-gated channels, and tumor incidence does not vary among KRAS-only injected embryos kept in dark, ambient light, or blue/red optogenetic exposure [data not shown]).

**Figure 3:**
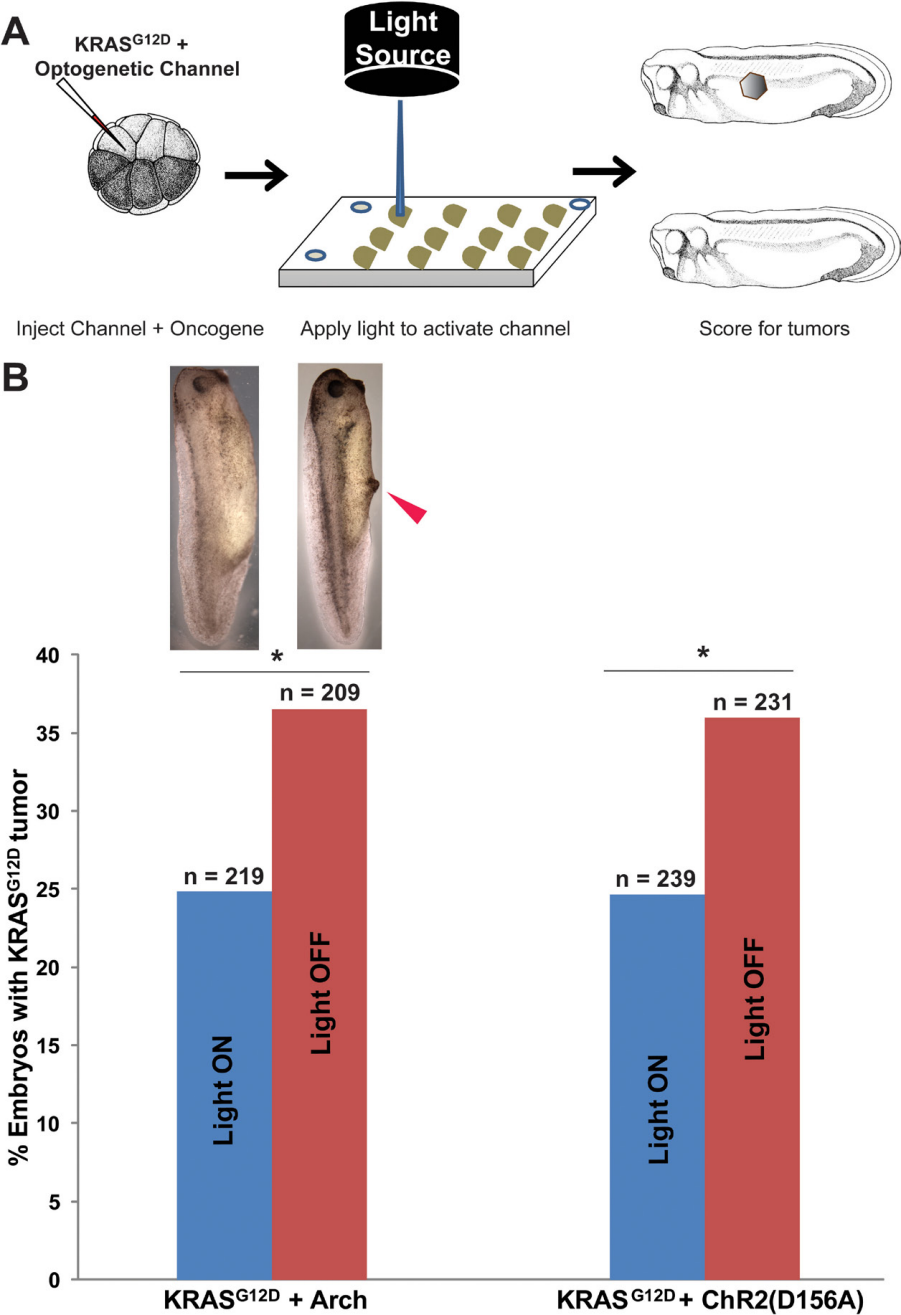
Expression of optogenetic channels Arch and ChR2^D156A^ prior to ITLS appearance suppresses the formation of *KRAS^G12D^* ITLSs (**A**) Schematic of experimental design for ITLS suppression: mRNAs for *KRAS^G12D^* and *ChR2^D156A^* or *Arch* were co-injected into a single blastomere of 16-cell stage embryos; between 4 and 28 hours post injection (a total exposure of 24 hours), injected embryos were exposed to 580 nm wavelength of light, 1 mw/mm^2^ irradiance (stimulating *Arch*) or 450 nm wavelength of light with 2.4 mw/mm^2^ irradiance (stimulating *ChR2^D156A^*). *Arch* and *ChR2^D156A^* experiments were done separately to test the hypothesis that ITLS suppression is due to changes in membrane voltage as opposed to channel or ion specific properties. Light-stimulated embryos were raised to stage 35 and scored for ITLSs to assess the efficacy of hyperpolarizing optogenetic channels as ITLS suppressing reagents. (**B**) The two different hyperpolarizing translocators – based on active pumping of H^+^ (*Arch*) and passive diffusion of positive, monovalent cations (*ChR2^D156A^*) – both resulted in suppression of ITLSs, demonstrating that this effect is likely due to a change in V_mem_ and is not tied to one channel protein. 32% and 31% fewer embryos with ITLS were observed among *Arch* and *ChR2^D156A^* injected embryos, respectively (*Arch*: c^2^ = 7.1, *p = 0.007; *ChR2^D156A^*: χ^2^ = 6.8, *p = 0.009. χ^2^ values are for comparisons of ITLS incidence in light stimulated versus un-stimulated embryos).

Consistent with our previous finding that hyperpolarization suppresses ITLS formation, our experimental data show that *Arch* activity significantly reduces the number of embryos that develop *KRAS^G12D^* ITLSs by 32% compared to *KRAS^G12D^*-expressing embryos lacking Arch (χ^2^ = 7.1, *p =* 0.007) (Figure 3B). Similarly, the activity of *ChR2^D156A^* was able to significantly lower *KRAS^G12D^* ITLS incidence by 31.4% (χ^2^ = 6.8, *p =* 0.009) (Figure 3B). Together, these data show that light-activated *Arch* and *ChR2^D156A^* can reduce ITLS formation by *KRAS^G12D^* in non-excitable tissues, suggesting that light-dependent activity of optogenetic reagents is a V_mem_-altering modality with efficacy in the suppression of ITLS formation.

### Light-initiated *ChR2^D156A^* activity normalizes fully developed *KRAS^G12D^* ITLSs

**Figure 4:**
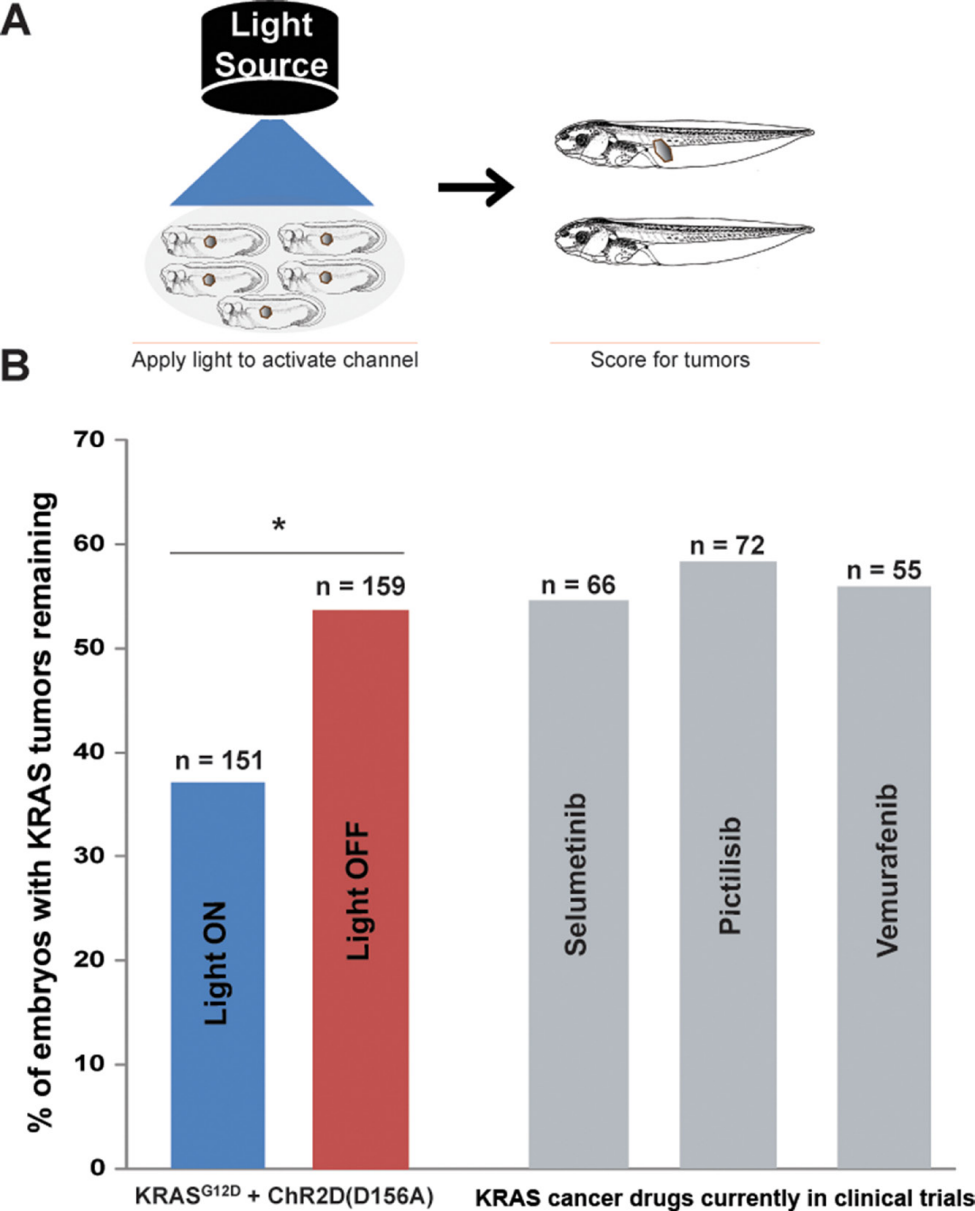
After ITLS formation, activation of *ChR2^D156A^* normalizes *KRAS^G12D^* ITLSs (**A**) Schematic of experimental design for ITLS normalization: *ChR2^D156A^* expressing embryos (stages 28–35) with *KRAS^G12D^* ITLSs were subjected to 450 nm wavelength of light with irradiance of 0.5 mw/mm^2^. Light-stimulated embryos were raised to stage 45–47 and scored for ITLSs to assess the ability of optogenetic V_mem_ modulation to normalize ITLSs. (**B**) Light activation of *ChR2^D156A^* in ITLSs (blue bar) resulted in 31% more embryos with normalized ITLSs compared to their un-stimulated counterparts (red bar) (χ^2^ = 8.6, *p = 0.003). By comparison, treatment with the highly-selective MEK 1 inhibitor (Selumetinib), a potent PI3K inhibitor (Pictillisib), or a potent inhibitor of oncogenic B-RAF kinase activity (Vemurafenib), resulted in the same *KRAS^G12D^* tumor incidence as in the unstimulated control.

We next tested the utility of optogenetics in the conversion of existing ITLSs into normal tissue. Similar to the suppression experiments, *ChR2^D156A^* was co-expressed with *KRAS^G12D^*; in these experiments, however, stimulation of the light-dependent channel was delayed until stages between 28–35 when ITLSs were fully developed. Tadpoles were then scored for presence or absence of ITLSs when they reached stages 45–47 (Figure 4A). Stimulation of *ChR2^D156A^*-expressing cells within the tumors resulted in 31% more embryos having normalized their tumors – compared to their injected but un-stimulated counterparts (χ^2^ = 8.6, *p = 0.003) (Figure 4B). These results demonstrate that *ChR2^D156A^* increases the number of oncogene-induced ITLSs that are normalized.

Moreover, optogenetic stimulation had a larger effect than several drugs considered promising anti-tumor therapies in human cancer medicine: the highly-selective MEK 1 inhibitor Selumetinib; the potent Pi3K inhibitor Pictillisib; and the inhibitor of oncogenic B-RAF kinase activity Vemurafenib (grey bars, Figure 4B).

### *ChR2^D156A^* activity regulates muscle marker expression in KRAS-induced ITLSs

**Figure 5:**
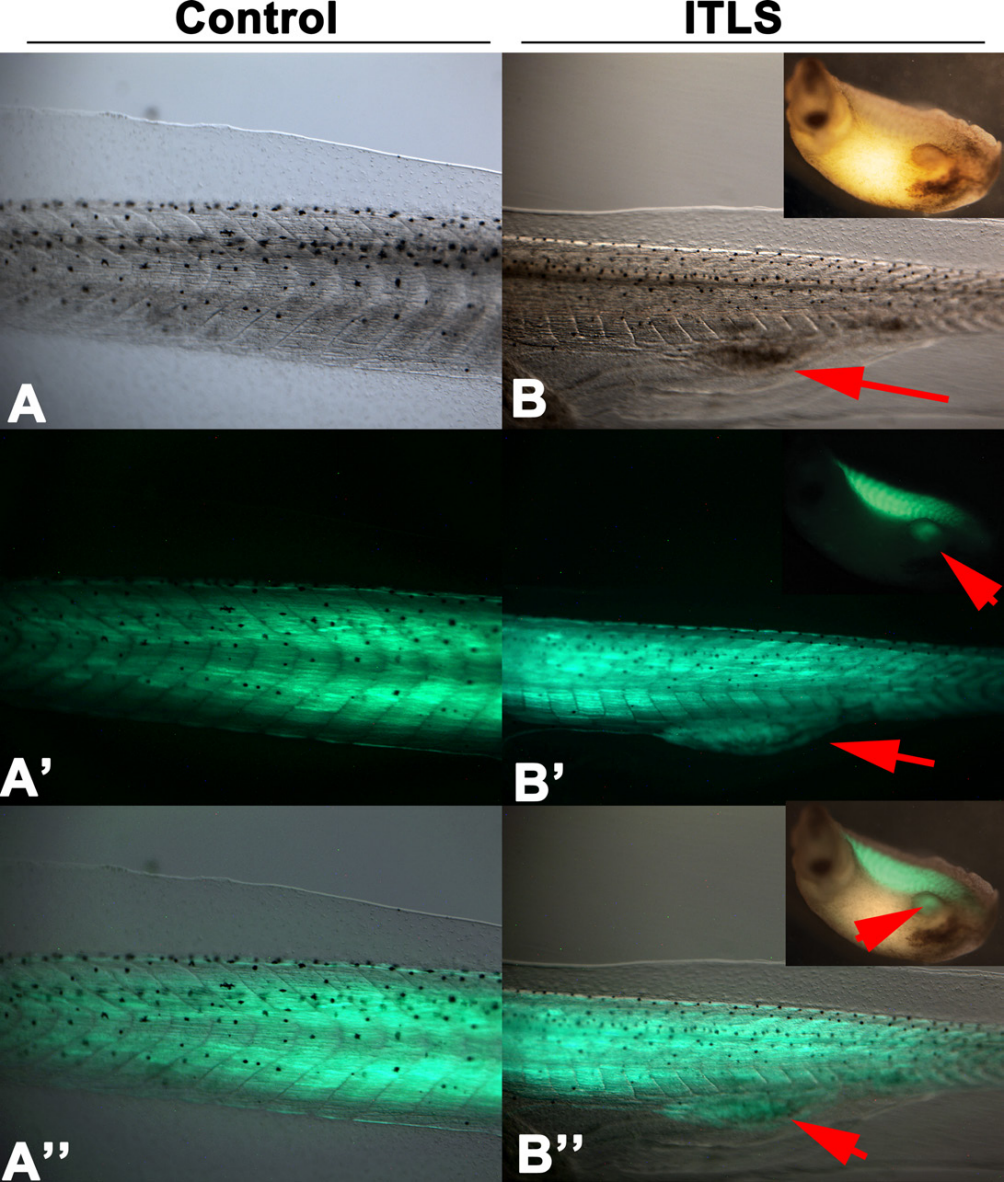
Muscle specific expression of GFP3 controlled by the cardiac actin (Car) promoter is present in *KRAS^G12D^ ITLS* Tadpoles injected with Tol2-CarPr-GFP3 [101] at the 2-cell stage display normal muscle specific localization of GFP3 within the somites (A, A′, A” showing transmitted light, GFP fluorescence, and both, respectively). In contrast, Tol2-CarPr-GFP3 tadpoles also injected with *KRAS^G12D^* reveal a strong GFP3 signal in ITLS's, confirming the presence of ectopic muscle in the KRAS-induced tumor-like structures.

**Figure 6:**
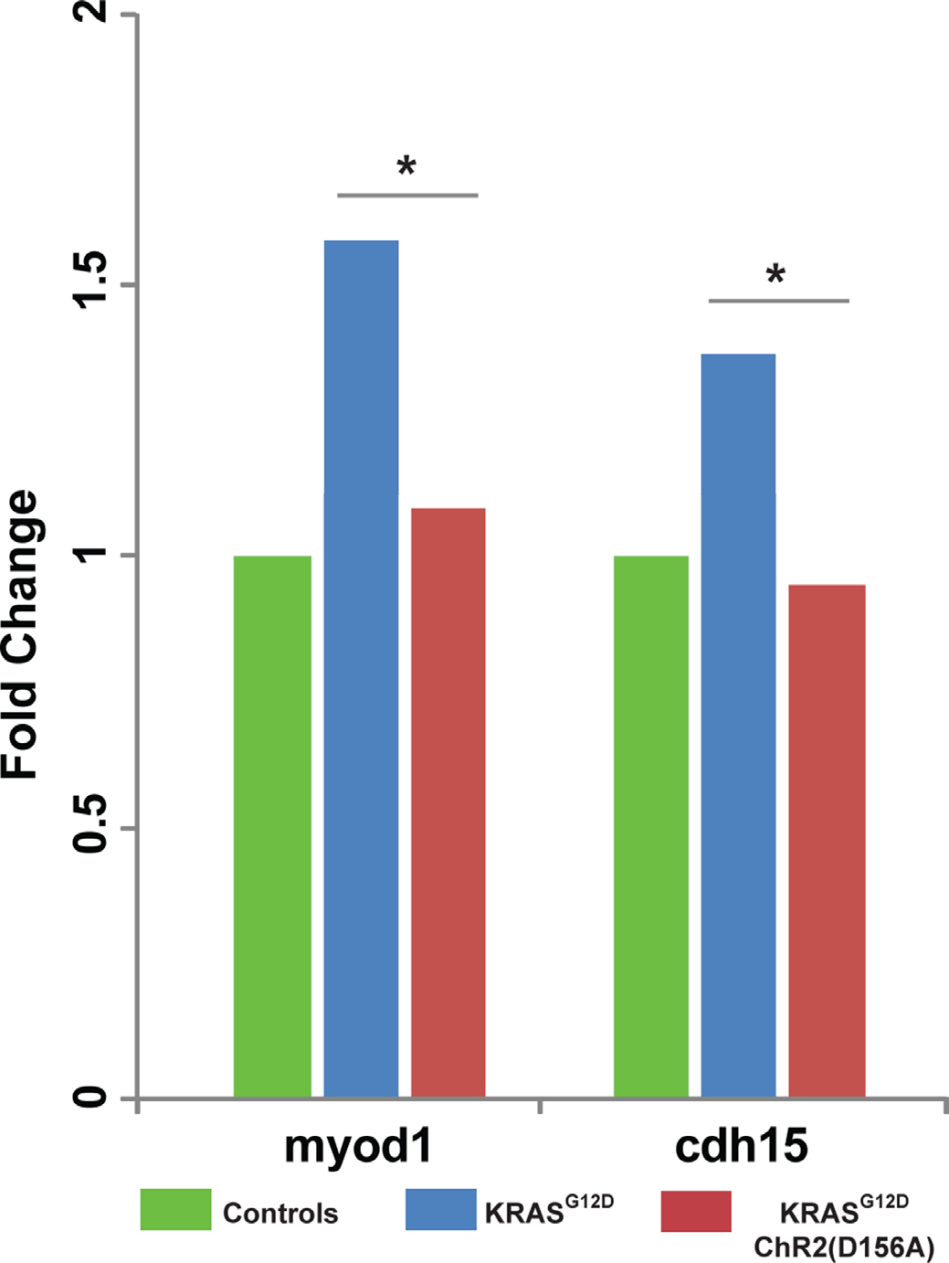
Light activation of *ChR2^D156A^* results in downregulated expression of ectopic muscle markers Human tumors associated with KRAS mutations express the muscle markers myod1 and cdh15. Using qPCR (see Materials and Methods for details), we found that expression of myod1 and cdh15 was likewise up-regulated in tadpoles with *KRAS^G12D^*-induced ITLSs. Moreover, activation of *ChR2^D156A^* in these tadpoles significantly lowered myod1 and cdh15 expressions, down to baseline levels (χ^2^ test compared to controls, *p < 0.01).

To determine whether there is a link between *ChR2^D156A^* activation and the expressions of tumor-specific transcriptional markers, we examined *chd15* (a satellite cell marker expressed in differentiating myoblasts) and *myod1* (a developing muscle cell marker expressed in newly formed somites and involved in differentiation). We chose these two markers because the *KRAS^G12D^* induced rhabdomyosarcoma arises from skeletal muscle tissue, and because these markers signal the presence of rhabdomyosarcoma in human samples [79–81]. As expected from comparison with *KRAS^G12D^*-induced human tumors, ITLSs observed growing in a transgenic line of animals that drive muscle-specific GFP fluorescence [82] revealed the presence of ectopic muscle cells (Figure 5). Using qPCR, we next found that both muscle markers were significantly up-regulated in tadpoles with *KRAS^G12D^* tumors (Figure 6, green bars) compared to uninjected controls (Figure 6, blue bars); however, the expressions of *myod1* and *cdh15* became normalized to control levels when *ChR2^D156A^* was activated in tadpoles (Figure 6, red bars). Taken together, these data show that optogenetic modulation of ion flux and resting potential reduces the expression of tumor markers as well as normalizes the tissue structure.

## DISCUSSION

Given the instructive role of bioelectric parameters in orchestrating cell behavior towards adaptive pattern formation [53, 83–85], our lab is interested in using modulation of resting potential *in vivo* as an approach to understanding misregulation of developmental signaling as occurs in cancer, birth defects, and other disease conditions. Previous work has shown that tumor-like structures induced in *Xenopus laevis* embryos by mammalian oncogenes (Figure 1A) exhibit many of the properties of tumors: overproliferation, expression of known tumor markers, attraction of vasculature, and histological disorganization [3, 40]. They also possess a depolarized potential relative to adjacent cells that are not part of the ITLS – a property long-known to be associated with cancer cells [24, 86], that was recently shown to be an instructive factor, not merely a read-out; actively preventing this depolarization can prevent the appearance of ITLSs [4]. In this study, we extend our ability to control tumorigenic pathways by expanding the use of optogenetics to this new system, and we exploit the temporal and cellular resolution afforded by light-gated ion channels to remove ITLSs after they have already been formed.

We have previously demonstrated the functionality of *Arch* in *Xenopus* embryonic and larval tissue: light activation of this hyperpolarizing H^+^ pump restores tail regeneration capability at non-regenerative stages [49]. Here, we show that light activation of another light-gated channel (*ChR2^D156A^*) also induces hyperpolarization in non-excitable *Xenopus* cells (Figure 1C). This differs from mammalian neurons where high Na^+^ concentration in the medium favors influx of the cation through channels, resulting in depolarization; *Xenopus* embryos grow in very-low extracellular Na^+^ medium, thus opening of cation channels results in hyperpolarization [87].

Channelrhodopsin variant D156A was chosen because of many optogenetic constructs tested [48] it caused the fewest unwanted side effects on embryogenesis. While ChR2 does exhibit some desensitization under illumination, this was not a problem in this application because high spiking rates are not used to encode information in this kind of developmental bioelectricity context [45]. Moreover, exposure of *ChR2^D156A^* was limited to 10 ms pulses, followed by 30s of darkness, i.e. a regimen than minimizes desensitization [88] and the shift away from a mixture of positive cations to primarily proton flux [89]. Thus, we are confident that stimulation of *ChR2^D156A^* led to the hyperpolarization we measured due to efflux of a mixture of positive cations. We found that the surface expression of *Arch* and *ChR2^D156A^* coupled with the non-toxic, high temporal-resolution light regimen, makes the optogenetic approach (Figure 2) suitable for targeting tumorigenesis *in vivo*.

We successfully exploited light-induced hyperpolarization of *Arch* expressing cells to decrease ITLS incidence among embryos expressing the *KRAS^G12D^* oncogene (Figure 3A, 3B). Importantly, compared to expressing constitutively-open hyperpolarizing channels, the light regimen was delivered for only 24 hours, was temporally more precise and convenient, and the technique is equally effective at suppressing ITLSs. To rule out any Arch H^+^-pump-specific ITLS suppression effects, we also employed light activation of the *ChR2^D156A^* non-specific cation channel in similar experiments (Figure 3A), thereby generating hyperpolarizing current by a different protein and a different mechanism, finding that passive cation efflux, like active H^+^-efflux, reduced the number of embryos with *KRAS^G12D^*-induced ITLSs (Figure 3B). The results obtained using the two distinct optogenetic channels that are not restricted to one type of ion flux, are consistent with V_mem_ *per se* being the instructive factor in regulating oncogene-mediated tumorigenesis [4, 10]. We also demonstrate for the first time that light-gated channels expressed in cells of tadpoles with KRAS ITLSs can be non-invasively activated for a duration sufficient to significantly increase the frequency of ITLS normalization, an exciting finding suggesting the possibility of remission-induction in addition to prevention (Figure 3, 4). Moreover, we show that the performance of this non-invasive optogenetic stimulation is superior to other anti-tumor agents we tested, including those that have been shown to be promising in human cancer medicine: Selumetinib, a highly-selective MEK 1 inhibitor; Pictillisib, a potent Pi3K inhibitor; and Vemurafenib, a potent inhibitor of oncogenic B-RAF kinase activity, (grey bars, Figure 4B). While these are the first data testing these compounds in the Xenopus tumor assay, we cannot rule out that subsequent research could identify a treatment regime that would allow these compounds to be more efficacious.

Our qPCR data (Figure 6) indicate that light-induced hyperpolarization of tumors also involves normalization of the expression of tumor markers, although it must be kept in mind that mRNA data do not necessarily reflect a linear correspondence to the presence of protein. Together, the data show that manipulation of bioelectric cell state, a powerful, tractable regulator of cancer cell normalization and reprogramming, is possible using optogenetics, thus introducing a new class of biomedical strategies for tumor treatment. The recent development of pharmacological approaches to render existing ion channels light-sensitive [90–92] suggests a next-generation approach that would not require introduction of transgenes (optogenetic channels) into the target tissue.

In summary, we report here the first use of optogenetics as a temporally precise regulator of V_mem_ to suppress and normalize oncogene-induced ITLSs. More broadly, this tool will advance the study of resting potential as another important component of the microenvironment that is so crucial for cancer initiation and progression [93–97]. Our implementation of light-gated bioelectrical signaling *in vivo* highlights another opportunity for the optogenetic toolbox to extend beyond excitable cells. Moreover, our data suggest a light-based therapeutics strategy that couples gene therapy with optogenetics to counteract tumorigenesis and promote regression *in vivo*.

## MATERIALS AND METHODS

### Animal husbandry

*Xenopus laevis* eggs were fertilized *in vitro*, and embryos were cultured according to standard protocols [98], in 0.1 × Modified Marc's Ringers (MMR; pH 7.8) with 0.1% Gentamicin. *Xenopus* embryos were housed at 14–18°C and staged according to Nieuwkoop and Faber [99]. All experimental procedures involving the use of animals for experimental purposes were approved by the Institutional Animal Care and Use Committees (IACUC) and Tufts University Department of Lab Animal Medicine (DLAM) under the protocol number M2014–79.

### Microinjection

Fertilized *Xenopus* embryos were transferred into mesh-bottomed dishes with 3% Ficoll and injected with capped, synthetic mRNAs (made using the Ambion Message Machine kit) dissolved in water at the stages indicated. The doses per cell were *KRAS^G12D^* [71] 40pg; *Arch* [69], 60pg; and *ChR2^D156A^* [72] 50pg. Two hours after injection, embryos were transferred into 0.75 × MMR for 45 minutes before they were washed and cultured in 0.1 × MMR until desired stage was reached. Injected embryos were stimulated with the appropriate wavelength of light and irradiance before or after ITLSs fully form (stages 28–35). Embryos were scored for the presence of ITLSs using bright field microscopy as described in [3, 41, 42].

### Light stimulation

During ITLS suppression experiments, an optogenetic set up (Figure 2) was used so that each embryo, in a microfluidic chip placed on a motorized stage, individually received a light regimen with the following parameters: *Arch* – 580 nm wavelength, 1 mw/mm^2^ irradiance, 500 ms on, 1.50s off; *Ch2R_(D156A)_* – 450 nm wavelength, 2.4 mw/mm^2^ irradiance, 10 ms on, 30s off. For normalization experiments involving Ch2R (D156A), a petridish of embryos with *KRAS^G12D^* ITLS were placed beneath an array of six LEDs delivering 450 nm of light with an irradiance of 0.5 mw/mm^2^.

### V_mem_ imaging

DiBAC_4_(3) (bis-(1, 3-dibutylbarbituric acid) trimethine oxonol) (DiBAC; Biotium, Inc, Hayward, CA, USA) was used to measure relative polarization. Light-stimulated embryos were transferred into a DiBAC_4_(3) solution (1.9 mM stock in DMSO used at 1:1000 in 0.1 × MMR), and imaged while still in the DiBAC_4_(3) solution. An Olympus BX-61 equipped with a Hamamatsu ORCA AG CCD camera, controlled by MetaMorph software, was used for imaging. DiBAC_4_(3) filters were: EX 470/20; BS 485; EM 517/23 (Chroma filter set 41001).

### RNA extraction and cDNA synthesis

RNA extraction was achieved using RNeasy Mini Kit (50) (QIAGEN 74104). Tadpoles (collected *n =* 10 per Eppendorf tube, three biological replicates) were put into 10 volumes of RNAlater solution. RNAlater-stabilized tissues were then removed from the reagent using forceps and placed into 2 ml Eppendorf tubes with 600 ul of B-ME containing Buffer RLT for disruption and homogenization. Disruption and homogenization of tissue was achieved using MICROSONTM XL 2000 ULTRASONIC LIQUID PROCESSOR. The lysate was then centrifuged for 3 minutes at full speed and the supernatant (lysate) was put into a new microcentrifuge tube. After adding and pipet-mixing 1 volume of 70% Ethanol into the lysate, 700 ul of the resultant sample was transferred to RNeasy spin column. Following the steps outlined in the RNeasy MiniHandbook 04/2006, we completed series of treatments with buffer RW1, RPE and RNase-free water to obtain total RNA. RNA yield and quality were assessed by spectrophotometry (ND-1000, NanoDrop) and gel electrophoresis, respectively, to assess integrity of 28S and 18S RNA.

Reverse transcription was performed using ThermoScript RT-PCR System (Life Technologies). Each *in vitro* reverse transcription reaction was performed using 1 μg of total RNA and 50 μg of oligo(dT)20 primers (Life Technologies). RNA and primers were mixed, denatured for 5 min at 65°C, and placed on ice before adding the reaction mix according to the manufacturer's instructions. Reverse transcription reaction was carried out at 50°C for 45 min. The reaction was terminated by incubating at 85°C for 5 min, followed by RNA degradation using 1 μg of RNase H for 20 min at 37°C. The complementary DNA (cDNA) was stored at −20°C until use. The quality and quantity of cDNA were validated using Advantage 2 PCR kit (Clontech) on cDNA samples using Orinithine Decarboxylase (ODC) primers.

### Quantitative real-time PCR

Primers were designed using Genius for myogenic differentiation 1 (Myod1) and myogenic differentiation 1 (Cdh15). ODC, a widely used endogenous control for Xenopus, was used to normalize target gene expression. The PCR specificity was verified by BLAST (http://blast.ncbi.nlm.nih.gov/Blast.cgi) using the National Center for Biotechnology Information X. laevis reference sequence. Desalted primers were obtained from Invitrogen by Life Technologies as follows: MyoD1-Forward CCGAGGGCAGTCCCTGTT; MydoD1-Reverse TGGGA CAGTTGAGTGCAGG; Cdh15-Forward ACAATCGT CCAGTGTTTGTGC; Cdh15-Reverse GTTCAGCATT GTCTGTCCTTGG. For each primer pair, standard curve primer analysis was performed using serial dilutions of cDNA from control embryos [1 (undiluted), 10−1, 10−2, 10−3]. Formation of primer-dimer and amplification specificity was assessed by efficiency and melt curve analysis. The cDNA from validated RNA was used to perform RT-qPCR assays. For each biological sample, three technical replicates were run in each RT-qPCR experiment. Each treatment contained five biological replicates. Triplicate negative controls lacking template were also run for each cDNA sample for each reaction. PCRs were assembled manually. Samples were prepared by adding 1 μl of cDNA (diluted 1:5 in ddH2O), 10 μl of 2 × Power SYBR Green PCR Master Mix (Applied Biosystems), and 0.5 μl of each primer (diluted to 10 μM) in a final volume of 20 μl. Reactions were incubated in 96-well MicroAmp Optical Reaction plates at 95°C for 10 min followed by 40 cycles at 95°C for 15 s and at 60°C for 1 min in a StepOnePlus qPCR instrument (Applied Biosystems). The RT-qPCR data were analyzed using the StepOne software v.2.3, and ΔΔCT values were calculated (Applied Biosystems). Fold change of target genes relative to the amount of the control gene ODC was calculated as 2^−ΔΔCT.

### Statistics

Following the appropriate light regimens, stimulated construct-expressing embryos were compared – for ITLS incidence – to their expressing but un-stimulated counterparts using a χ^2^ test (α = 0.01).

### Pharmacological agents

The stages selected for the antineoplastic drugs mirror those of the light treatment. Embryos with ITLS between St. 28–35 were selected, treated with the drugs, and scored for ITLS between St. 45–47. Concentration of the drugs and their effects on control embryos vs embryos with ITLS are given below. Following the manufacture's instruction we prepared stock solutions in DMSO of Selumetinib, Pictilisib, and Vemurafenib in concentration of 100 mM, 50 mM, and 25 mM, respectively. Embryos were then exposed in 0.1 × MMR for the stages indicated to: Selumetinib −100 nMm, Pictillisib −1 μM, and Vemurafenib −1 μM. All three compounds were obtained from Selleckchem.
